# Ethnomedicinal plants used for digestive system disorders by the Karen of northern Thailand

**DOI:** 10.1186/s13002-015-0011-9

**Published:** 2015-04-09

**Authors:** Kornkanok Tangjitman, Chalobol Wongsawad, Kaweesin Kamwong, Treetip Sukkho, Chusie Trisonthi

**Affiliations:** Department of Biology, Faculty of Science, Chiang Mai University, Huaykaew Road, Chiang Mai, 50200 Thailand; Royal Park Rajapruek, Mae-hea, Muang, Chiang Mai, 50200 Thailand

**Keywords:** Ethnobotany, Traditional knowledge, Gastrointestinal, Chiang Mai, Pharmacology, Toxicology

## Abstract

**Background:**

Digestive system disorders have a substantial effect on worldwide morbidity and mortality rates, including in Thailand, where the majority of the rural areas have a lack of proper sanitation and awareness about disease prevention. This has led to the prevalence of different types of digestive diseases. Karen people in Thailand still use medicinal plants as first aid remedies in treating these diseases. Therefore, this study aimed at documenting the plants used to cure and prevent different types of digestive system disorders by Karen people of Chiang Mai Province, northern Thailand.

**Methods:**

Ethnomedicinal data were collected from six key informants and 172 non-specialist informants regarding their traditional knowledge of medicinal plants. Quantitative approaches were used to determine Use Value (UV), Informant Consensus Factor (ICF) and Fidelity Level (FL) values.

**Results:**

The study revealed that 36 medicinal plant species belonging to 31 genera and 24 families were used to treat digestive system disorders. The most prevalent plant families were Zingiberaceae (6 species), Euphorbiaceae (4 species) and Fabaceae (4 species). Leaves were the most commonly used plant part accounting for 32.6% of the plants, followed by the bark (18.6%). About 60% of the administrations were given orally by potion (60%) and consumption as food was also indicated (14%). The highest ICF values were recorded for carminative disorders, stomachaches, geographic tongue, constipation, appetite stimulants and food poisoning (1.00 each) indicating the best agreement among the informants knowledge of medicinal plants that were used to treat aliments in these categories. The highest fidelity level values were recorded for *Punica granatum* (100.00), *Psidium guajava* (95.45), and *Gymnopetalum integrifolium* (90.91) showing conformity of knowledge on species with the best healing potential.

**Conclusion:**

Medicinal plants still play an important role among Karen culture. The present information on these medicinal plants, which have high UV and FL values, may serve as the baseline data to initiate further research for the discovery of new compounds and the biological activities of these potential plant remedies. Further research on these plants may provide some important clues for the development of new drugs for the treatment of digestive system diseases.

## Introduction

There are a wide number of digestive system disorders, which impose a substantial influence on morbidity and mortality rates, worldwide. The World Health Organization (WHO) [1] reported that digestive system disorders, particularly diarrhea, was the fifth leading cause of global mortality, as approximately 100 million people died worldwide in 2012 from these types of disorders. Moreover, in South-East Asia, diarrhea has been the cause of 10% of deaths among children below the age of 5 years.

For the last couple of years, there has been a global trend in the renewal of interest in a traditional system of treatments. Ethnomedicinal plant studies have become of particular interest and have become increasingly more valuable in the development of health care and conservation programs in different parts of the world [2]. The WHO has recognized the role of traditional medicine in the primary health care system [3]. In developing countries, medicinal plants continue to be a main source of medication. It has been estimated that approximately 88% of the inhabitants of underdeveloped countries rely mainly on traditional medicine for their primary form of medicinal health care [4].

Thailand has a rich population of ethnic people who still maintain a traditional knowledge of medicinal plants that are used in the treatment of illnesses [5]. Studies of several ethnomedicinal plants have been carried out among several ethnic groups in Thailand. However, there has been no comprehensive study of the medicinal plants used to treat digestive system disorders in Thailand. Digestive system disorders were identified as the third highest cause of morbidity among Thai people in 2010 [6]. More than 1 million people have appealed to the public health system for the treatment of these diseases. Besides, Jansongduang et al. [7] revealed that people who typically reside in remote areas especially hill tribe people in northern Thailand likely drink water from forest streams without any antiseptics and used the same water for bathing, raising livestock, and sewage disposal. These practices result in poor water quality and often lead to digestive system problems. Moreover, previous ethnobotanical studies in northern Thailand showed that digestive diseases had the highest number medicinal plants recorded compared with other illness categories and most hill tribe people had experience curing these diseases with medicinal plants [7-10]. This reflects that digestive system disorders are also important morbidity among Thai hill tribe people.

This study documents the traditional medicinal plants that are used for digestive system disorders by the Karen which comprise the largest hill tribe in Thailand [11]. The Karen originated in Tibet and had migrated to other parts of Southeast Asia, particularly Myanmar [12]. From the 18th century onwards they began to cross the Salween River and moved into Thailand, where they settled in the high mountains of Chiang Mai, Mae Hong Son and Lamphun provinces, as well as other areas. In 2003, the Karen people constituted 48% of the total hill tribe population in the region with a population more than 430,000 Karens in Thailand [11]. As they typically reside in the mountain areas, the Karen people have limited access to public healthcare systems. They have therefore accumulated a rich experience related to preventing and treating diseases with herbal remedies, and they have developed a distinctive knowledge of traditional medicine. This traditional knowledge has been handed down from one generation to the next by spoken word and through lifestyle. Most Karen villagers still maintain traditional knowledge of medicinal plants that are used for first aid remedies and to treat simple ailments [10].

## Materials and methods

### Study area

**Figure 1:**
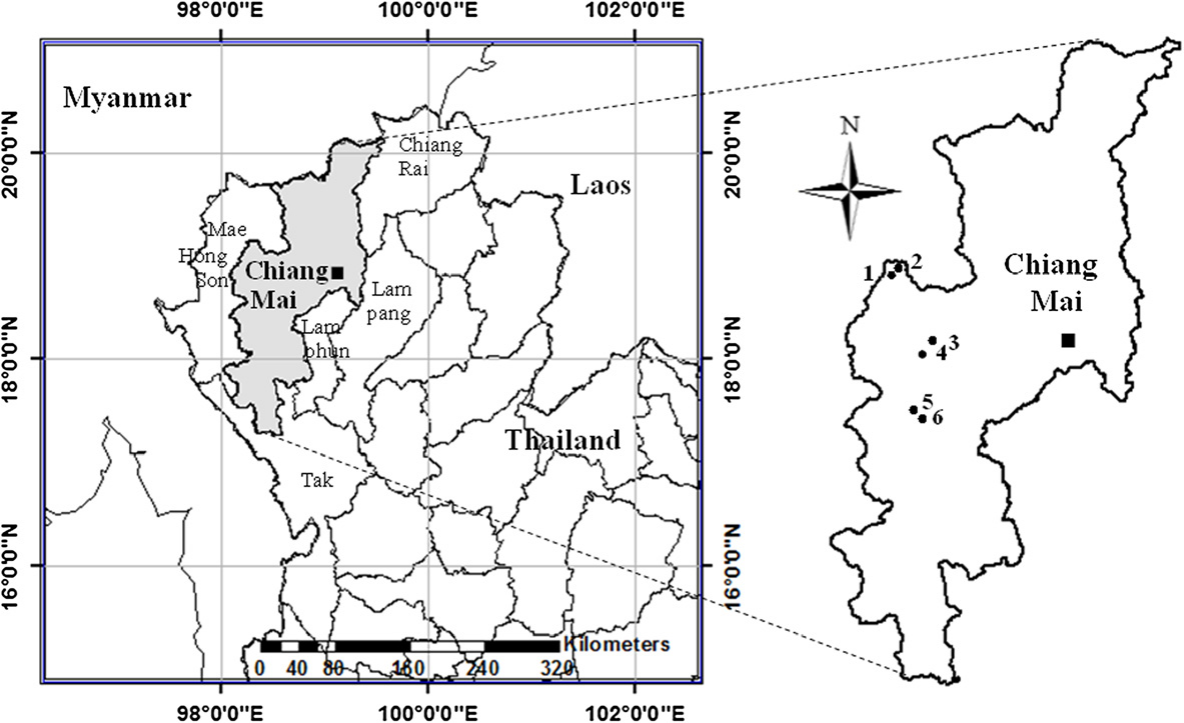
**Map of study areas in Chiang Mai province, Thailand: (1) Kew Pong; (2) San Muang; (3) Mai Lan Kam; (4) Huay Hea; (5) Huay Pu Ling; (6) Mai Sa Wan.**

Data were collected in Chiang Mai province, northern Thailand (Figure 1). Chiang Mai province is surrounded by high mountain ranges and covers an area of approximately 20,107 km^2^ [13]. Forest area covers 17,640 km^2^ (72.01%) of Chiang Mai’s total area. The major types of forests in Chiang Mai are mixed deciduous forests, tropical evergreen forests and dry dipterocarp forests. Several national parks are also located in the province (Doi Inthanon, Doi Suthep-Pui, Mae Ping, Sri Lanna, Huay Nam Dang, Mae Phang and Chiang Dao). Six Karen villages (Huay Hea, Mai Lan Kam, Kew Pong, San Muang, Mai Sa Wan and Huay Pu Ling) were selected as study sites. These villages are located at 746, 692, 1,010, 1,050, 1,190 and 1,050 m.l.s., respectively and they are surrounded by natural forests. There are 14 households in Huay Hea, 45 in Mai Lan Kam, 49 in Kew Pong, 51 in San Muang, 18 Mai Sa Wan and 32 in Huay Pu Ling. They typically consume upland rice and supplement their meals with vegetables and animal products for their diet. The villagers derive their main monetary income through the sale of forest products, livestock and as labor in northern Thai fields. Their economic status is generally considered to be poor. The Karen society is matriarchal. Each household contains only one or two generations. Most Karen people in Kew Pong, San Muang, Mai Sa Wan and Huay Pu Ling are Christian whereas those of Huay Hea and Mai Lan Kam consider themselves Buddhist.

### Data collection

To collect plants and associated ethnomedicinal information relating to digestive system disorders from the Karen, field trips were conducted between 2006 and 2011. Initial contacts were made to the village headmen, to whom we explained the purpose and techniques of the proposed research. Subsequently the headmen explained the purpose and methods of the study to the villagers who gave their informed consent for the publication of this report and any accompanying images. The information on medicinal plants was gathered through interviews, guided tours, and participative observation in homegardens, cultivated fields and nearby forests. The plants used were indentified (local name), photographed and samples were collected for the preparation of herbarium specimens, which were deposited at the Faculty of Science, Chiang Mai University and the Queen Sirikit Botanical Garden Herbarium (QBG), Chiang Mai, Thailand. Plant identification was based largely on taxonomic literature, such as through the use of references entitled the Flora of Thailand, the Flora of China and the Flora of Java.

Semi-structured interviews were conducted to determine the overall prevalence of medicinal plant knowledge. This was done from October 2011 through April 2012 with 172 non-specialist informants (80 males and 92 females, aged 13–92 years). All medicinal plant species data obtained from the key informant were prepared for the interview. During the interview, plant pictures and the Karen plant names were shown to the informant. Questions were asked individually concerning the actual use of the medicinal plants used in the therapy of digestive system disorders, along with questions about what plant part was used, the mode of preparation, and the route of administration. The semi-structured interviews were conducted in Thai in the presence of a translator when the informants were not able to communicate in the Thai language.

### Data analysis

#### Use value (UV)

The relative importance was calculated employing the use value [14], a quantitative measure for the relative importance of a given species known locally:

$$ \mathrm{U}\mathrm{V}=\sum U/\mathrm{n} $$

where *U* is the number of use-reports cited by each informant for a given species and n refers to the total number of informants. Use values are high when there are many use-reports for a plant, implying that the plant is important, and the approach is zero (0) when there are few reports related to its use. The use value, however, does not distinguish whether a plant is used for single or multiple purposes.

#### Informant consensus factor (ICF)

To test homogeneity of knowledge, the informant consensus factor was used [15]:

$$ \mathrm{I}\mathrm{C}\mathrm{F}=\left({N}_{\mathrm{ur}}\hbox{--} {N}_{\mathrm{t}}\right)/\left({N}_{\mathrm{ur}}-1\right) $$

where *N*_ur_ refers to the number of use-reports for a particular use category and *N*_t_ refers to the number of taxa used for a particular use category by all informants. A lower ICF value (near 0) indicates the informants’ disagreement of using a particular plant to treat a particular ailment category, and a higher ICF value (approach 1) is indicative of using relatively few plants by the informants in the treatment of a particular ailment category [16].

#### Fidelity level (FL)

Because many plant species may be used in the same use category, it is interesting to determine the most preferred species used in the treatment of a particular ailment, which can be done with the Fidelity Level (FL) of Friedman et al. [17]:

$$ \mathrm{F}\mathrm{L}\left(\%\right)=\left( Np/N\right)*100 $$

where *N*p is the number of use-reports cited for a given species for a particular use category and *N* is the total number of use-reports cited for any given species. High FLs (near 100%) are obtained for plants for which almost all use reports refer to the same way of using a given plant whereas low FLs are obtained for plants that are used for many different purposes.

## Results

### Medicinal plants and traditional uses

A total of 36 plants belonging to 31 genera and 24 families were recorded as being used by the Karen in treating different types of digestive system disorders (Table 1). The reported plant families include Zingiberaceae (6 species), Euphorbiaceae (4 species), Fabaceae (4 species), Musaceae (2 species), Acanthaceae, Apiaceae, Acoraceae, Asparagaceae, Celastraceae, Cucurbitaceae, Dilleniaceae, Flacourtiaceae, Juglandaceae, Lamiaceae, Lauraceae, Leeaceae, Melastomataceae, Myrsinaceae, Myrtaceae, Ochnaceae, Fabaceae, Poaceae, Punicaceae, Rhamnaceae and Rubiaceae (1 species each).

**Table 1:** **Medicinal plants used to treat digestive system disorders by the Karen people of northern Thailand**

**Scientific name (Voucher no.)**	**Family**	**Karen name**	**Part used**	**Preparation**	**Route of administration**	**Application**	**Use value**
*Acorus calamus* L. (K. Kamwong106)	Acoraceae	Por bue lah	rhizome	non, decoction	eaten as food, potion	stomach ache	0.14
*Asparagus filicinus* Buch.-Ham. ex D.Don (K. Kamwong097)	Asparagaceae	Ya su mae	root	decoction	potion, bath	gastric ulcer, jaundice hemorrhoid, flatulence	0.10
*Boesenbergia rotunda* (L.) Mansf. (K. Kamwong099)	Zingiberaceae	Por sa raw	rhizome	decoction	potion	flatulence	0.39
*Cassytha filiformis* L. (K. Kamwong073)	Lauraceae	Se kruy po	all	decoction	potion, bath	jaundice	0.21
*Celastrus paniculatus* Willd. (K. Kamwong031)	Celastraceae	Ti si bler	bark	decoction	potion	diarrhea	0.08
*Centella asiatica* (L.) Urb. (T. Sukkho157)	Apiaceae	Chuy po co la do	leaf	non	eaten as food	gastric ulcer, diarrhea	0.09
*Croton kongensis* Gagnep. (K. Kamwong032)	Euphorbiaceae	Sa ko wa	leaf	decoction	potion, bath	gastric ulcer, jaundice, diarrhea	0.30
*Croton robustus* Kurz (K. Kamwong033)	Euphorbiaceae	Sa ko wa sui	leaf, bark	decoction	bath	jaundice	0.21
*Curcuma longa* L. (T. Sukkho071)	Zingiberaceae	Si yaw	rhizome	non, decoction	eaten as food, potion	gastric ulcer, flatulence	0.56
*Dendrocalamus strictus* (Roxb.) Nees (K. Kamwong284)	Poaceae	Wa mee	leaf	decoction	potion, bath	jaundice	0.36
*Dillenia pentagyna* Roxb. (K. Kamwong204)	Dilleniaceae	Kho tee	bark	decoction	potion	gastric ulcer	0.52
*Embelia sessiliflora* Kurz (T. Sukkho006)	Myrsinaceae	Blea blor	fruit	non	eaten as food	laxative	0.09
*Engelhardtia spicata* Blume var. *colebrookeana* (Lindl. ex Wall.) Kuntze (K. Kamwong208)	Juglandaceae	Klue por	bark	decoction	potion	gastric ulcer	0.59
*Ensete glaucum* (Roxb.) Cheesman (K. Kamwong020)	Musaceae	Ya pa la	leaf sheaf	decoction	potion	diarrhea, food poisoning	0.23
*Euphorbia heterophylla* L. (T. Sukkho055)	Euphorbiaceae	Nor bo lo bell	leaf, latex	decoction, non	potion, liniment	laxative, mouth ulcer	0.47
*Euphorbia hirta* L. (K. Kamwong272)	Euphorbiaceae	-	all	decoction	potion	gastric ulcer	0.43
*Flacourtia jangomas* (Lour.) Raeusch. (K.Kamwong241)	Flacourtiaceae	Ser pae	bark	non, decoction	hold in mouth, potion	toothache, gastric ulcer, diarrhea	0.14
*Flemingia macrophylla* (Willd.) Merr. (K. Kamwong209)	Fabaceae	Chor ae go bor	bark	decoction	potion	jaundice	0.28
*Gmelina arborea* Roxb. (K. Kamwong122)	Lamiaceae	Ker ma	bark, flower	decoction	potion	gastric ulcer, laxative	0.11
*Gymnopetalum integrifolium* Kurz. (T. Sukkho166)	Cucurbitaceae	Se do kwaw mee	leaf, stem	decoction	potion, bath	jaundice, flatulence	0.19
*Kaempferia parviflora* Wall. ex Baker (T. Sukkho039)	Zingiberaceae	Por sue	rhizome	decoction	potion	gastric ulcer, flatulence	0.17
*Leea indica* (Burm. f.) Merr. (K. Kamwong135)	Leeaceae	Na tor kor	root, stem	decoction	potion	diarrhea, hemorrhoid, gastric ulcer	0.14
*Melastoma malabathricum* L. (K.Tang054)	Melastomataceae	Se la play	fruit	non	hold in mouth	mouth ulcer, geographic tongue	0.37
*Musa sapientum* L. (K. Kamwong021)	Musaceae	Si kuy	fruit	non	eaten as food	diarrhea	0.34
*Mussaenda sanderiana* Ridl. (K. Kamwong034)	Rubiaceae	Por jor kaw pe	root, leaf	decoction	hold in mouth bath, potion	toothache, jaundice, appetite stimulant	0.17
*Ochna integerrima* (Lour.) Merr. (K. Kamwong028)	Ochnaceae	Ti si bor	leaf	decoction	potion, bath	constipation, jaundice, gastric ulcer, diarrhea	0.25
*Psidium guajava* L. (K. Kamwong115)	Myrtaceae	Ma kwuy	young leaf	decoction	potion	diarrhea	0.61
*Punica granatum* L. (T. Sukkho148)	Punicaceae	Chor pa lea	young leaf	decoction	potion	diarrhea	0.71
*Senna alata* (L.) Roxb. (K. Kamwong168)	Fabaceae	Ya la mer pa do	leaf	decoction	potion	laxative	0.49
*Senna occidentalis* (L.) Link (K. Kamwong183)	Fabaceae	Ya la mer	leaf	decoction	potion	laxative	0.59
*Tamarindus indica* L. (K. Kamwong093)	Fabaceae	Sa mor klae	fruit	non	eaten as food	laxative	0.26
*Thunbergia laurifolia* Lindl. (K. Kamwong083)	Acanthaceae	Jaw law lee der	leaf, stem	decoction	potion, bath	gastric ulcer diarrhea, jaundice	0.16
*Zingiber montanum* (J.König) Link ex A.Dietr. (K. Kamwong236)	Zingiberaceae	Blae ko bor	rhizome	decoction, non	potion, eaten as food	flatulence, gastric ulcer	0.72
*Zingiber officinale* Roscoe (K. Kamwong319)	Zingiberaceae	Sa ae	rhizome	decoction	potion	diarrhea, flatulence, gastric ulcer	0.30
*Zingiber ottensii* Valeton (K. Kamwong166)	Zingiberaceae	Blae ko sue	rhizome	decoction	potion	flatulence, carminative	0.74
*Ziziphus cambodiana* Pierre (K. Kamwong064)	Rhamnaceae	Ri co mae	bark	decoction	potion, bath	gastric ulcer, hemorrhoid, jaundice	0.16

### Plant part used, method of preparation, and route of administration

Among the different plant parts used — the leaf (32.6%) was the most frequently used plant part, followed by the bark (18.6%), the rhizome (16.3%), fruit (9.3%), the root (7.0%), the stem (7.0%), the whole plant (4.7%), the flower (2.3%) and the latex (2.3%). Herbal medicines were prepared in the form of decoction (83.8%), as well as those that were described as non-prepared (16.2%). The modes of administration were potion (60.0%), bath (18.0%), eaten as food (14.0%), held in mouth (6.0%) and applied as liniment (2.0%).

### Use records and aliments

A total 902 actual use records were registered among the interview with non-specialist informants. These belong to 14 different aliments (Table 2). The ailment for which there was the most frequently reported was diarrhea, which accounted for slightly more than 23% of all use records while flatulence (22%), laxative (19%), gastric ulcer (19%) and jaundice (10%) were also common.

**Table 2:** **Category of digestive system disorders and their Informant Consensus Factor (ICF)**

**Category of digestive system disorder**	**Number of use reports (** ***Nr*** **)**	**Number of taxa (** ***Nt*** **)**	**ICF**
Diarrhea	209	12	0.95
Flatulence	201	8	0.97
Laxative	171	6	0.97
Gastric ulcer	168	14	0.92
Jaundice	92	11	0.89
Mouth ulcer	21	2	0.95
Geographic tongue	6	1	1.00
Constipation	2	1	1.00
Stomachache	7	1	1.00
Appetite stimulant	2	1	1.00
Food poisoning	2	1	1.00
Carminative	15	1	1.00
Toothache	3	2	0.50
Hemorrhoid	3	3	0.00

### Plant use values, informant consensus factor and fidelity levels

The High Use Value was recorded for selected species, such as *Zingiber ottensii* (0.74), *Zingiber montanum* (0.72), *Punica granatum* (0.71), *Psidium guajava* (0.61), *Senna occidentalis* (0.59), *Curcuma longa* (0.56) and *Dillenia pentagyna* (0.52) (Table 1). High UV levels indicate high numbers of use reports by the informants for a particular plant.

The informant’s consensus factor (ICF) was found to range between 0.00 and 1.00 (Table 2). The disease categories with the highest use reports were for carminative disorders, stomachaches, geographic tongue, constipation, as an appetite stimulant and for food poisoning (1.00 each), followed by flatulence and as a laxative (0.97 each), for diarrhea and mouth ulcers (0.95 each), gastric ulcers (0.92), jaundice (0.89), toothaches (0.50) and hemorrhoids (0.00).

The highest Fidelity Level (FL) for the plants used by the Karen was recorded as *Punica granatum* (100.00), followed by *Psidium guajava* (95.45), *Gymnopetalum integrifolium* (90.91), *Zingiber montanum* (90.20), *Senna occidentalis* (87.18), *Dillenia pentagyna* (84.62), *Zingiber ottensii* (81.75), *Musa sapientum* (81.58), *Engelhardtia spicata* var. *colebrookeana* (81.25), *Melastoma malabathricum* (76.92), *Dendrocalamus strictus* (75.00), *Euphorbia heterophylla* (72.22), *Curcuma longa* (70.63) and *Senna alata* (70.59) (Table 3).

**Table 3:** **Fidelity Level (FL) values for medicinal plants used by the Karen**

**Category**	**Most preferred species used against digestive disorders (FL(%))**
Diarrhea	*Punica granatum* (100.00), *Psidium guajava* (95.45), *Musa sapientum* (81.58), *Leea indica* (50.44), *Ensete glaucum* (50.00), *Celastrus paniculatus* (46.15), *Ochna integerrima* (33.33)
Gastric ulcer	*Dillenia pentagyna* (84.62), *Engelhardtia spicata* var. *colebrookeana* (81.25), *Curcuma longa* (70.63), *Croton kongensis* (42.31), *Ziziphus cambodiana* (36.36)
Flatulence	*Zingiber montanum* (90.20), *Zingiber ottensii* (81.75), *Boesenbergia rotunda* (65.00), *Kaempferia parviflora* (31.82)
Laxative	*Senna occidentalis* (87.18), *Euphorbia heterophylla* (72.22), *Senna alata* (70.59), *Tamarindus indica* (62.75)
Jaundice	*Gymnopetalum integrifolium* (90.91), *Dendrocalamus strictus* (75.00), *Croton robustus* (56.25), *Flemingia macrophylla* (46.15), *Mussaenda sanderiana* (44.19)
Mouth ulcer	*Melastoma malabathricum* (76.92)
Geographic tongue	*Melastoma malabathricum* (23.08)
Constipation	*Ochna integerrima* (2.38)
Stomachache	*Acorus calamus* (6.09)
Appetite stimulant	*Mussaenda sanderiana* (4.65)
Food poisoning	*Ensete glaucum* (7.14)
Carminative	*Zingiber ottensii* (10.95)
Toothache	*Mussaenda sanderiana* (4.65)
Hemorrhoid	*Ziziphus cambodiana* (9.09)

### Toxicity of species used medicinally

Toxicity data was available for only 18 of the 36 species. Table 4 shows general toxicity studies that have been previously recorded in evaluating the biological activities of these plants. Twelve species were reported to have a toxic effect on animals (*Acorus calamus*, *Cassytha filiformis*, *Celastrus paniculatus*, *Euphorbia heterophylla*, *Euphorbia hirta*, *Flemingia macrophylla*, *Kaempferia parviflora*, *Senna alata*, *Senna occidentalis*, *Thunbergia laurifolia* and *Zingiber montanum*), while six plants (*Centella asiatica*, *Curcuma longa*, *Gmelina arborea*, *Psidium guajava*, *Punica granatum* and *Tamarindus indica*) were reported to have no toxic effects on humans or animals.

**Table 4:** **Literatures reporting on toxicity studies for medicinal plants used by the Karen**

**Scientific name**	**Toxic effect**
*Acorus calamus* L.	acute toxicity in mice, LD_50_ = 221 g/kg [44]
*Cassytha filiformis* L.	acute toxicity in mice, LD_50_ = 625.8 g/kg [45]
*Celastrus paniculatus* Willd.	hyperactivity and loss of righting reflex in rat [46]
*Euphorbia heterophylla* L.	increase leucopaenia in rat [47]
*Euphorbia hirta* L.	leucocytosis, dullness, anorexia, stairy haircoat and 20% mortality in rat [47]
*Flemingia macrophylla* (Willd.) Merr.	severe hypoglycemia followed by death within 24 hour after administration to mice [48]
*Kaempferia parviflora* Wall. ex Baker	hepatotoxic to rat [49]
*Senna alata* (L.) Roxb.	decrease hemoglobin and erythrocyte (RBC) count values in rats [36]
*Senna occidentalis* (L.) Link	intestinal disturbance in long-term used in rats [50]
*Thunbergia laurifolia* Lindl.	decrease red blood cell in male mice [51]
*Zingiber montanum* (J.König) Link ex A.Dietr.	acute toxicity in mice, LD_50_ = 80 g/kg [52]
*Zingiber officinale* Roscoe	embryo toxic to pregnant rats [53]
*Centella asiatica* (L.) Urb.	no toxic effect in mice [54]
*Curcuma longa* L.	no toxic effect in human [55]
*Gmelina arborea* Roxb.	no toxic effect in rodents [56]
*Psidium guajava* L*.*	no toxic effect in mice [57]
*Punica granatum* L.	no toxic effect in rats [58]
*Tamarindus indica* L.	no toxic effect in mice [59]

## Discussion

### Medicinal plant use

The uses of 36 plant species used to treat 14 different aliments, by key and non-specialist informants, were reported in this study. The plants used most prevalently were from the family Zingiberaceae, which had six species. The Karen people prefer cultivating the Zingiberaceae plant for their personal consumption in their homegardens or in their fieldwork. Some of the most common dishes consumed by the Karen involve chili sauces and curry. The plants used in these dishes are usually cooked and the vegetables are sometimes part of the side dishes. Therefore, most Karen people are familiar with these plants and use them as food as well as for medicinal purposes.

As measured by the number of plant(s) per aliment, diarrhea was correlated with the highest number of plant species recorded (12 species) and also the highest number of uses recorded (209 uses). This might reflect the fact that diarrhea is quite prevalent in terms of morbidity among the Karen people. Most Karen villages are located in mountainous areas, which are geographically remote areas. Unsafe water supplies and inadequate levels of sanitation and hygiene may increase the transmission of diarrhea among the Karen people.

Leaves were the plant part that was used the most in the preparation of remedies by the Karen people, as compared to other parts. Many studies conducted elsewhere in northern Thailand also showed the dominance of the leaf in the preparation of remedies [8,9,18]. Leaves are the main photosynthetic organ in plants and are considered to be a key component of the natural pharmacy for the synthesis of many active constituents, particularly those that are more pharmacologically active against certain diseases [19]. Gathering leaves could be promoted as a sustainable practice, since in most cases at least, a number of leaves are left on the parent plant which then allows the plants to carry on their life functions [20].

In this study, herbal remedies were found to be largely prepared by decoction and were administered mainly orally by potions (60%) which were in agreement with the results of other studies conducted in northern Thailand [9,18,21]. Moreover, the results also revealed that almost all of the surveyed species are used singly as mono-herbal recipes with a specific part of the plant used for a particular disease.

The ICF values varied from 0.00 to 1.00 (Table 2). The highest recorded ICF values (0.89-1.00) indicated the best level of agreement among the informants in terms of the use of the medicinal plant species reported to be used for treating carminative disorders, geographic tongue, stomachaches, constipation, as well as an appetite stimulant, for food poisoning (1.00 each), flatulence, as a laxative (0.97 each), for diarrhea, mouth ulcers (0.95 each), gastric ulcers (0.92), and jaundice (0.89). However, hemorrhoids indicated the lowest value (0.00). According to Gazzaneo et al. [16] high ICF values are important in the identification of plants of particular interest in the search for bioactive compounds.

This study reported the highest fidelity level values for *Punica granatum* (100.00), *Psidium guajava* (95.45) and *Musa sapientum* (81.58) against diarrhea; *Dillenia pentagyna* (84.62), *Engelhardtia spicata* var. *colebrookeana* (81.25) and *Curcuma longa* (70.63) against gastric ulcers; *Zingiber montanum* (90.20) and *Zingiber ottensii* (81.75) against gastric ulcers; *Senna occidentalis* (87.18), *Euphorbia heterophylla* (72.22) and *Senna alata* (70.59) as a laxative; *Gymnopetalum integrifolium* (90.91), *Dendrocalamus strictus* (75.00) against jaundice and *Melastoma malabathricum* (76.92) against mouth ulcers. These medicinal plants could be considered a clue for the high healing potential of these plants against the corresponding diseases. Plants with the highest fidelity level values could also be targeted for further phytochemical investigations in order to identify the bioactive components that are responsible for their high healing potential.

### Ethnomedicinal relevance

Digestive system disorders are one of the most common types of ailments affecting humans. Several ethnomedicinal studies revealed that the use of medicinal plants by traditional people against digestive system disorders is a common practice throughout the world. These illnesses were the most important usage categories in many different countries and areas, such as in Ethiopia [22], Brazil [23], the Peruvian Andes [24] and Bolivia [25]. Interestingly, thirty-three (93%) of the studied plants were found to have been used for treating digestive system disorders in other ethnomedicinal investigations in different parts of the world (Table 5). Moreover, twenty-three (64%) of the surveyed plants shared similar use aliments with other studies. The repetitive usage of these plant species used by the Karen people may serve as an indication of their effectiveness and show a high value of medicinal plant knowledge for treating digestive system disorders.

**Table 5:** **Literature study of the surveyed medicinal plants and their relevant ethnomedicinal uses, pharmacological studies and known chemical constituents**

**Scientific name**	**Relevant ethnobotanical citations**	**Relevant pharmacological citations**	**Known chemical constituents**
*Acorus calamus* L.	Stomachache*, carminative [60]	antidiarrhoeal activity [61]	asarones [62]
*Asparagus filicinus* Buch.-Ham. ex D.Don	stomachache [60], diarrhea [63]	NA	steroidal saponins, oligospirostanosides, oligofurostanosides, aspafilioside [64]
*Boesenbergia rotunda* (L.) Mansf.	flutulence*, indigestion [65]	antiulcer activity [66], hepatoprotective activity [67]	boesenbergin, cardamonin, pinostrobin, pinocembrin, alpinetin [68]
*Cassytha filiformis* L.	liver disease [69] dysentery, gastric ulcer [29]	NA	aporphine alkaloids, cassyformine, filiformine [70]
*Celastrus paniculatus* Willd.	laxative 71]	NA	malkanguniol [71]
*Centella asiatica* (L.) Urb.	gastric ulcer* [72], diarrhea* [73]	ulcer protective effect** [74]	centelloids, pentacyclic triterpenoid, polyacetylenes, asiaticosides [75]
*Croton kongensis* Gagnep.	acute gastroenteritis*[76]	NA	NA
*Croton robustus* Kurz	NA	NA	trachyloban-19-oic acid, trachyloban-19-ol, poilaneic acid [77]
*Curcuma longa* L.	Carminative [78]	antiulcer activity**, hepatoprotective activity [40]	curcuminoid [79], zingiberene, sesquiterpenes, α-phellandrene [78]
*Dendrocalamus strictus* (Roxb.) Nees	dysentery [80]	NA	NA
*Dillenia pentagyna* Roxb.	diarrhea [81], jaundice [82]	NA	betulinic acid, naringenin 7-galactosyl, dihedral quercetin 5-glactoside [83]
*Embelia sessiliflora* Kurz	diarrhea [62]	NA	NA
*Engelhardtia spicata* Blume var. *colebrookeana* (Lindl. ex Wall.) Kuntze	mouth ulcer [21], diarrhea [62]	NA	Engelhardtione, oleanolic acid [84]
*Ensete glaucum* (Roxb.) Cheesman	gastritis, constipation [9], food poisoning* [60]	NA	NA
*Euphorbia heterophylla* L.	laxative*, constipation [85]	NA	stigmasterol, stigmasterol glucoside, benzoic acid, 4 – hydroxyl benzoic acid [85]
*Euphorbia hirta* L.	vomitting [86], diarrhea [73]	antidiarrhoeal activity [87]	quercitrin, myricitrin, Afzelin [88]
*Flacourtia jangomas* (Lour.) Raeusch.	toothache* [89], diarrhea* [90] liver disease [91]	antidiarrhoeal activity** [92]	phenolics, tannins, terpenoids [93]
*Flemingia macrophylla* (Willd.) Merr.	indigestive, flatulence [90]	hepatoprotective activity** [39]	fleminone, flemiphyllin [94]
*Gmelina arborea* Roxb.	diarrhea [95] stomach-hepatic diseases* [96]	antidiarrhoeal activity [97], hepatoprotective activity [98]	monoacylated, diacylated, triacylated iridoid glycosides [90]
*Gymnopetalum integrifolium* Kurz.	NA	NA	cucurbitacin [99]
*Kaempferia parviflora* Wall. ex Baker	gastric ulcer* [100]	antiulcer activity** [42]	methoxyflavones (5,7,4′ trimethoxyflavone, 5,7-dimethoxyflavone) [100]
*Leea indica* (Burm. f.) Merr.	diarrhea* [69]	NA	flavonids, leucoanthocyanidins, galic acid, amorphous froth forming acid [101]
*Melastoma malabathricum* L.	diarrhea, dysentery [102], toothache [103]	antidiarrhoeal activity [102]	ellagic acid, anthocyanin, nobotannin B [103]
*Musa sapientum* L.	gastric ulcer [104], diarrhea*, dysentery [105]	antidiarrhoeal activity* [32], antiulcer activity [106]	alkaloids, pectins, flavonoids, catecholamines, acyl steryl glycosides [105]
*Mussaenda sanderiana* Ridl.	laxative, toothache* [90]	NA	NA
*Ochna integerrima* (Lour.) Merr.	jaundice*, mouth ulcer [21] digestive tonic [107]	NA	ochnaflavone, dihydroochnaflavone, lophirone, calodenone [107]
*Psidium guajava* L.	diarrhea* [108]	antidiarrhoeal activity** [57]	tannins, polyphenolic compounds, triterpenoid, guajanoic acid [31]
*Punica granatum* L.	diarrhea* [109]	antidiarrhoeal activity** [26]	punicalagins, ellagic acid, tannin, anthocyanins [30]
*Senna alata* (L.) Roxb.	laxative*, colon cleanser [110]	stimulant laxative activities** and againt costipation [36]	anthraquinoes, phenols, tannins, saponins, flavonoids [34]
*Senna occidentalis* (L.) Link	laxative* [111], liver disease [112] gastrointestinal disease [113]	stimulant laxative activities** [50]	anthaquinones [35]
*Tamarindus indica* L.	laxative* [114], gastric ulcer [112]	stimulant laxative activities** [37] antiulcer activity [115]	tamarindienal, tartaric acid, malic and tartaric acids potassium acid [37]
*Thunbergia laurifolia* Lindl.	stomachache, carminative [9]	hepatoprotective effects** [116]	iridoid glucosides, delphinidin, apigenin [52]
*Zingiber montanum* (J.König) Link ex A.Dietr.	flatulence*, carminative [5]	antiulcer activity* [41]	terpinen-4-ol, phenylbutenoids, zerumbone [52]
*Zingiber officinale* Roscoe	flatulence* [113]	prokinetic activity [117] hepatoprotective activity [118]	gingerols, shogaols [119]
*Zingiber ottensii* Valeton	jaundice [120]	NA	zerumbone, terpinen-4-ol, p-cymene [52]
*Ziziphus cambodiana* Pierre	NA	NA	triterpenes, saponins, cyclopeptide, alkaloids [121]

*ethnomedicinal knowledge in other ethnic groups which have similar usage to medicinal plants used by the Karen.

**pharmacological study which is relevant to the medicinal plants used by the Karen.

NA: data not available.

### Pharmacology relevance and chemical constitute

Several investigators have reported on the pharmacological relevance of the plants used in digestive system disorders. In the present study, 19 medicinal plants were found to be pharmacologically active against digestive system disorders (Table 5). The highest numbers of pharmacological activity reported were for antiulcer activity (15 species), antidiarrhoeal activity (8 species), hepatoprotective activity (8 species), stimulant laxative activity (2 species) and prokinetic activity (1 species). Moreover, the ethnomedicinal uses of 12 plants were similar with the pharmacological activities reported. Therefore, these investigations might confirm that some medicinal plants have a potential effect on treating digestive system disorders.

The important medicinal plants for treating diarrhoeal diseases were *Punica granatum* (UV = 0.71; FL = 100.00), *Psidium guajava* (UV = 0.61; FL = 95.45), *Musa sapientum* (UV = 0.34; FL = 81.58), *Leea indica* (UV = 0.14; FL = 50.44), *Ensete glaucum* (UV = 0.23; FL = 50.00). Several investigations revealed that tannins and other polyphenolic compounds, such as coumarins, flavonoids, triterpenoids, saponins, and a host of other plant secondary metabolites possess antidiarrhoeal properties [26-28]. Particular tannins are responsible for protein denaturation and for producing the protein tannate, which reduces secretions from intestinal mucosa [29]. Studies on the phytochemical properties showed that the crude extracts of *Punica granatum* seed [30] and *Psidium guajava* leaves [31] contain numerous tannins. Therefore, these plants may produce antisecretory activity and antidiarrhoeal activity in animals. Another pharmacological study [32] revealed that pectins, which are found in the cell wall and in intracellular substances in many fruits, had therapeutic effects on treating diarrhea. It was also reported that green bananas (*Musa sapientum*) were rich in pectins and could significantly reduce diarrhea in children.

The second highest use recorded among the Karen people was for flatulence. Six of eight plants used for treating this illness were from the Zingiberaceae family (*Boesenbergia rotunda* (UV = 0.39; FL = 65.00), *Curcuma longa* (UV = 0.56; FL = 5.56), *Kaempferia parviflora* (UV = 0.17; FL = 31.82), *Zingiber montanum* (UV = 0.72; FL = 90.20), *Zingiber officinale* (UV = 0.30; FL = 18.42) and *Zingiber ottensii* (UV = 0.34; FL = 81.75)). A biological study revealed that the active constituents in the essential oils, such as the gingerols in zingiberaceae plants, inhibited a multiplication of the colon bacteria that ferment undigested carbohydrates causing flatulence [33].

The most prevalent plants used as laxatives were *Senna occidentalis* (UV = 0.59; FL = 87.18), *Senna alata* (UV = 0.49; FL = 70.59), *Euphorbia heterophylla* (UV = 0.47; FL = 72.22), and *Tamarindus indica* (UV = 0.26; FL = 62.75). The important phytochemical constituents in *Senna* were anthraquinones [34,35], which are known to have stimulant laxative properties [36]. Moreover, these phytochemicals are present in various drugs used in Europe. Another study on the laxative properties of medicinal plants determined the significance of *Tamarindus indica* [37]. It was reported that the tartaric acid, malic acid and potassium acid in this plant were major constituents inducing laxative activity.

Jaundice is an ailment characterized by a yellowish pigmentation of the skin, the conjunctival membranes over the sclerae (the whites of the eyes), and other mucous membranes [38]. It is often seen in liver diseases such as hepatitis or liver cancer. The use of medicinal plants to treat jaundice was also recorded as high in this study. The important plants used for treating this ailment were *Dendrocalamus strictus* (UV = 0.36; FL = 75.00), *Flemingia macrophylla* (UV = 0.28; FL = 46.15), *Croton robustus* (UV = 0.21; FL = 56.25), *Gymnopetalum integrifolium* (UV = 0.19; FL = 90.91), and *Mussaenda sanderiana* (UV = 0.17; FL = 44.19). Interestingly, most medicinal plants used for treating jaundice were administrated by bath and potion. This reflects the culturally herbal administration for treating specific aliments by the Karen people. Moreover, a pharmacological study revealed that the aqueous extract of *Flemingia macrophylla* had a hepatoprotective effect against liver damage in rats [39].

A high use for the treatment of gastric ulcers was also recorded (168 uses) among the Karen people. Several plants were used for treating this disease, including *Engelhardtia spicata* var. *colebrookeana* (UV = 0.59; FL = 81.25), *Curcuma longa* (UV = 0.56; FL = 70.63), *Dillenia pentagyna* (UV = 0.52; FL = 84.62), *Croton kongensis* (UV = 0.30; FL = 42.31), *Ziziphus cambodiana* (UV = 0.16; FL = 36.36), *Kaempferia parviflora* (UV = 0.17; FL = 13.64), *Zingiber montanum* (UV = 0.72; FL = 13.64). A pharmacological study on *Curcuma longa* [40] found that curcumin, which was the active constituent in this plant, had a beneficial effect on the stomach. It could block indomethacin, ethanol in stress-induced gastric ulcers and could also prevent pylorus-ligation-induced acid secretion in rats. Moreover, the antiulcer activity study on *Zingiber montanum* reported that zerumbone, which was the important phytochemical property of this plant, showed potent cytoprotective and antiulcerogenic effects against hydrochloric acid (HCl) induced gastric ulceration in mice [41]. Another pharmacological investigation involved *Kaempferia parviflora* [42]. Its ethanolic extract could increase gastric mucus secretions, which were related to the preservation of ulcer-damaged tissue.

Overall, several plants displayed effective biological activities against different digestive aliments. However, a literature search found that some medicinal plants used by the Karen people have not been included in any pharmacological studies on the digestive system. Therefore, it is of significant interest to investigate the biological research of some of the Karen people’s medicinal plants used for the treatment of digestive system disorders.

### Toxicity of medicinal plants

A literature investigation found that 18 of the 36 species named had been previously exposed to a toxicology study (Table 4). Six species (*Centella asiatica*, *Curcuma longa*, *Gmelina arborea*, *Psidium guajava*, *Punica granatum* and *Tamarindus indica*) had no toxic effect on animals, whereas 12 species showed a toxic effect. The different toxic effects recorded after administrating medicinal plants were found in the digestive system (*Flemingia macrophylla*, *Kaempferia parviflora* and *Senna occidentals*), the blood cells and the circulatory system (*Euphorbia heterophylla*, *Euphorbia hirta*, *Senna alata* and *Thunbergia laurifolia*), the nervous system (*Celastrus paniculatus*), and the reproductive system (*Zingiber officinale*). Moreover, a study on the acute toxicity of these treatments on mice found that *Zingiber montanum* had the highest toxicity level (LD_50_ = 80 g/kg).

Most Karen people believe that medicinal plants do not produce any side effects. They are also cheap and locally available. However, natural products may also contain a few harmful ingredients as secondary metabolites [43] which may produce perilous side effects. Therefore, medicinal plants must be taken in the proper amounts and long-term administration must be avoided for the optimal health and well-being of the patient.

## Conclusion

Digestive system disorders have a high prevalence in terms of the morbidity rate among Thai people. This is also considered to be true worldwide, particularly among ethnic people who likely have inadequate access to hygienic levels of sanitation, which may increase the transmission of digestive diseases. The study of medicinal plants among the Karen people of northern Thailand has reported that 36 species were commonly used against digestive system disorders. A literature investigation found that several surveyed plants had similar usage with other ethnic groups in different areas throughout the world. Moreover, the pharmacological studies of some of the medicinal plants could confirm that these plants are considered effective in treating digestive diseases. However, some medicinal plants, which were reported to have high UV and FL values, still require further pharmacological research for the discovery of new compounds and biological activities of these potential medicinal plants. There were certain toxic effects that were found to have been associated with some of these plants. Therefore, herbal remedies should be taken carefully in order to avoid any potential side effects that may occur through utilizing these medicinal plants.
